# Research on the Development of an Inland Lake Bathymetry Estimation Model Based on Multispectral Data

**DOI:** 10.3390/s25072236

**Published:** 2025-04-02

**Authors:** Junzhen Meng, Yunfei Wang, Wenkai Liu, Xiaoquan Yang, Peipei He

**Affiliations:** College of Surveying and Feo-Informatics, North China University of Water Resources and Electric Power, Zhengzhou 450046, China; wangyunfei202303@163.com (Y.W.); liuwenkai@ncwu.edu.cn (W.L.); 17739004720@163.com (X.Y.); hepei@ncwu.edu.cn (P.H.)

**Keywords:** bathymetric estimation, reflectivity extraction methods, remote sensing indices, random forests, underwater 3D topographic maps

## Abstract

Lakes play a crucial role in regional economic development and ecological construction. The variation in lake water depth has a direct impact on local economic activities, such as agriculture, livestock farming, and fisheries, as well as the stability of hydrological conditions and water ecology. In response to the lack of unified evaluation in the application of remote sensing water-depth estimation models for inland lakes, this study systematically compares the performance of numerical models and machine learning models for water-depth estimation in inland lakes. A machine learning-based water-depth estimation model construction methodology suitable for inland lakes is proposed. This study introduces an innovative approach by integrating machine learning techniques with multispectral remote sensing data, improving the accuracy and applicability of water-depth estimation models for inland lakes. The results show the following: (1) The machine learning models based on random forest (RF), BP neural networks (BP), and AdaBoost demonstrate better performance (R^2^ = 0.88, 0.72, and 0.61; MAE = 0.12 m, 0.24 m, and 0.31 m; RMSE = 0.32 m, 0.48 m, and 0.57 m) compared to the multi-band logarithmic ratio (MLR) model (R^2^ = 0.59; MAE = 0.32 m; RMSE = 0.58 m); (2) the machine learning water-depth estimation model constructed based on this methodology exhibits improved precision (R^2^ = 0.92, 0.89, and 0.80; MAE = 0.11 m, 0.17 m, and 0.25 m; RMSE = 0.25 m, 0.30 m, and 0.41 m). This suggests that the methodology is more suitable for the estimation of water depth in medium- and small-sized lakes; (3) The machine learning model developed in this study, combined with multispectral remote sensing imagery, achieves the accuracy required for the evaluation of water depths for practical water resources. This model enables the rapid acquisition of high-precision underwater three-dimensional topographic maps, providing more accurate and timely hydrological data support for lake water resource management.

## 1. Introduction

Lakes, as an essential component of surface water resources, play an irreplaceable role in ecosystem support, water resource storage, climate regulation, environmental protection, and socio-economic values [1].

Traditional methods of wate-depth measurement, such as single-beam [2,3] and multi-beam echo sounding [4], can provide high-accuracy depth information. However, they are limited by factors such as the draft of the survey vessel; difficulties in field measurements; and the complexity of data processing, high labor costs, and low efficiency. As a result, these methods struggle to map the water-depth distribution of large areas in a short period. Satellite remote sensing technology, with its advantages in efficiency, wide coverage, near-real-time updates, and high resolution, effectively compensates for the shortcomings of traditional methods [5]. Polcyn et al. [6] successfully realized the remote measurement of shallow water depths for the first time by measuring wave reflection changes. Building on this, Lyzenga et al. [7] proposed a two-layer flow theory model to address the limitations of the band ratio model for water-depth estimation, and they used multispectral aerial imagery for shallow sea area depth estimation for the first time, enabling the practical application of remote sensing water-depth estimation techniques. With the advancement of research, multispectral data water-depth estimation models can generally be classified into theoretical models, statistical models, and semi-theoretical, semi-empirical models [8,9,10,11]. Each model has its advantages and disadvantages. Theoretical models [12] allow for the in-depth exploration of the relationship between radiative energy and water depth, but they are cumbersome and involve many parameters. Statistical models [13], while simpler, are more susceptible to environmental influences. Semi-theoretical, semi-empirical models [14,15] simplify parameter quantification but have limited applicability. To overcome the limitations of these models, this study selects the MLR model, Adaboost model, BP model, and RF model for water-depth estimation. Among them, the MLR model [16] utilizes the relationship between multispectral reflectance in remote sensing images and water depth, effectively eliminating the influence of bottom substrates and improving estimation accuracy; the Adaboost model [17] is an ensemble learning method that combines multiple weak classifiers to construct a strong classifier, with excellent generalization ability and overfitting prevention; the BP model [18], based on the error backpropagation algorithm and multi-layer feedforward neural network, has self-learning and self-adaptive capabilities, enabling flexibility in different scenarios; the RF model [19,20,21], utilizing an ensemble of decision trees, boasts strong noise resistance and the ability to process high-dimensional data. The advantages of these models provide diverse, high-precision solutions for water-depth estimation.

In addition, current research on water-depth estimation based on machine learning algorithms mainly focuses on the construction of estimation models and the comparison of performance among various machine learning models. However, studies on the impact of different band combinations and reflectance extraction methods on model accuracy during the water-depth estimation process are relatively scarce [22,23,24]. To address this gap, this paper first systematically analyzes the accuracy performance of MLR models and machine learning models under GDAL and Rasterio environments. Based on this analysis, we propose a new inland lake water-depth estimation model construction methodology. This methodology utilizes Rasterio to extract reflectance values from image bands and optimizes the water-depth estimation model by integrating remote sensing indices. The goal is to develop a remote sensing model that can accurately invert inland lake water-depth data and, based on this model, rapidly generate high-precision underwater three-dimensional topographic maps, thereby providing more accurate and timely hydrological data support for lake water resource management.

## 2. Data and Preprocessing

### 2.1. Study Area

**Figure 1:**
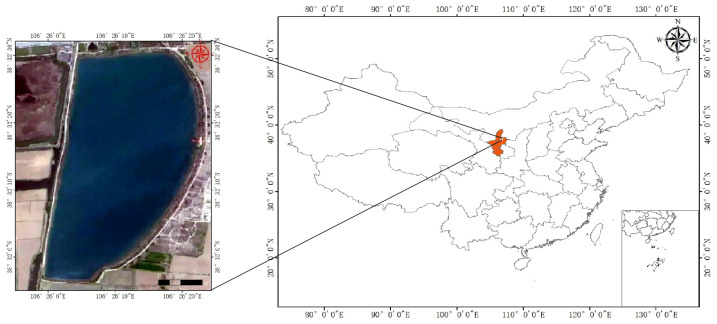
Schematic diagram of the study area.

As shown in Figure 1, the study area is located in Panchang Village and Jiangnan Village in Jinguizhen, 3 km east of Helan County in Yinchuan City. The area covers approximately 0.5 km^2^, with geographic coordinates of 106.40° E and 38.44° N. The lake is oval in shape, with its axis running in the north–south direction. The water quality around the lake is clear and transparent, indicating oligotrophic conditions, and the lakebed primarily consists of predominantly sandy substrates mixed with aquatic plant debris, with some aquatic plant debris. The water depth is moderate and stable, making it an important site for studying lake ecology and environmental protection. Due to its location in a rural area and its proximity to the Yellow River basin, the primary water supply for the lake comes from the Yellow River and rainfall.

### 2.2. Bathymetric Data

In the Qingshui Lake study area, an “unmanned vessel + single-beam sonar + RTK” system was used to accurately collect water depth data, enabling real-time measurement through acoustic technology. By integrating GNSS information, a three-dimensional underwater topographic map was created. Data collection was conducted on 29 June 2022, covering water depths ranging from 0 m to 7 m. After data preprocessing and filtering, 27,808 valid water-depth points were obtained and divided into a 0.6 training set and a 0.4 testing set to validate the model’s performance, thus laying the foundation for subsequent water-depth inversion research. Additionally, the specific parameters and accuracy information of the main measuring instruments are provided in Table 1.

### 2.3. Satellite Imagery Data

The imagery used in this study was sourced from the Landsat 9 satellite, available through the Geospatial Data Cloud, which provides reflectance data across seven bands, as shown in Table 2. The image was acquired on 5 April 2022 and meets the L2-level quality standard. It has undergone preprocessing, including radiometric calibration, geometric correction, and atmospheric correction. Additionally, a review of weather conditions in Helan County, Yinchuan City, from 5 April 2022 to 29 June 2022, through online sources, revealed that no extreme rainfall or drought events occurred during this period, with minimal weather impact. Furthermore, no significant human interference was observed in the Qingshui Lake area during this time. Therefore, the remote sensing image from 5 April 2022, was selected for water-depth inversion in this study. In this study, the preprocessing of satellite imagery primarily involved two core steps: land–water separation and geometric registration. The process is as follows: Water–land separation was performed using the modified normalized difference water index (MNDWI) [25]. This index effectively highlights water bodies while suppressing land features. The specific formula is as follows:

(1) MNDWI=(Green+SWIR)/(Green−SWIR)

where Green represents the green band, and SWIR represents the shortwave infrared band. This formula allows for the accurate extraction of water bodies from satellite imagery.

In the geometric registration process, two independent methods were utilized, both implemented in the Python 3.10 environment: one combining Rasterio with Numpy and Pandas and the other combining GDAL, Numpy, Pyproj, and Pandas. These methods aimed to align the measured water-depth points with their corresponding pixels in the remote sensing image within a geographic space. Subsequently, the reflectance values of the pixels corresponding to the measured water-depth points were extracted from the remote sensing image.

## 3. Bathymetric Estimation Modeling

### 3.1. Bathymetric Estimation Factor Selection

The inversion of the depth of the water is a complex process and its precision is influenced by multiple factors, including characteristics of the water body, environmental interference, and the quality of the remote sensing data [26,27,28,29]. The errors in water-depth inversion related to remote sensing data are mainly due to the quality of remote sensing imagery and the image processing workflow. In addition, different reflectance extraction methods and inversion factors can also significantly impact inversion results. Therefore, to accurately invert the depth of the water, it is essential to select the appropriate inversion factors and suitable reflectance extraction methods.

Firstly, a Pearson correlation analysis was conducted between the reflectance data extracted using GDAL and Rasterio and the measured water depth, as shown in Table 3. The correlation coefficients between the reflectance values of each band and the measured water depth exhibited a similar trend for both methods: an initial increase followed by a decrease. However, the correlation coefficients obtained using the GDAL method are generally higher than those from Rasterio, with an average difference of 0.48. Based on the correlation coefficient scale ($\left|r\right|$ 0.8 indicates high correlation, 0.5–0.8 indicates moderate correlation, 0.3–0.5 indicates low correlation, and $\left|r\right|$ < 0.3 indicates weak correlation), under the Rasterio method, bands B4, B5, B6, and B7 show low correlations with the water depth, while under the GDAL method, the correlation between B1 (low correlation), B2, and B3 (moderate correlation) and the water depth is higher. Therefore, in terms of correlations, GDAL is more suitable for reflectance extraction in water-depth inversion. However, the correlation coefficient only reflects linear relationships, and further analyses are needed to assess nonlinear relationships. Secondly, the reflectance values of the remote sensing image bands and remote sensing indices extracted under the Rasterio environment were selected as inversion factors to optimize the model’s input structure and analyze the impact of remote sensing indices on the water-depth inversion results in order to improve the accuracy and reliability of water-depth inversions. The correlation coefficients are detailed in Table 4.

### 3.2. Methodology

#### 3.2.1. Multi-Band Logarithmic Ratio Model

The MLR model is an extension of the two-band model, suitable for variations in water types within a single category. Even in cases with sufficient spectral data, it can also handle scenarios where both water and bottom types change within a single scene [30]. The formula is as follows:

(2) $$\begin{array}{cc} Z & =m_{0}+m_{1}\times \frac{\ln\left[n\times R_{w}\left(\lambda_{2}\right)\right]}{\ln\left[n\times R_{w}\left(\lambda_{1}\right)\right]}+\frac{\ln\left[n\times R_{w}\left(\lambda_{3}\right)\right]}{\ln\left[n\times R_{w}\left(\lambda_{2}\right)\right]}+\cdots +\frac{\ln\left[n\times R_{w}\left(\lambda_{n}\right)\right]}{\ln\left[n\times R_{w}\left(\lambda_{n-1}\right)\right]} \end{array}$$

#### 3.2.2. Machine Learning Model

Based on the Python platform, machine learning models for water-depth inversion were specifically developed using the RF [31], AdaBoost [32], and BP (back propagation) neural network algorithms [33].

### 3.3. Precision Evaluation

This study uses three metrics, mean absolute error (MAE), root mean square error (RMSE), and coefficient of determination (R^2^), to evaluate the performance of the model. The formulas are as follows:

(3) R2=1−∑i=1nyi−y^i2∑i=1nyi−y¯i2

(4) RMSE=∑i=1nyi−y^i2n

(5) MAE=∑i=1nyi−y^in

where $y_{i}$ represents the measured depth, yi^ describes the estimated depth, $\overset{¯}{y_{i}}$ represents the mean value of the measured depths, and n indicates the number of input data.

## 4. Results

This study proposes a water-depth inversion model construction scheme for inland lakes based on the reflectance values of image bands and remote sensing indices extracted using Rasterio. The model is used to perform water-depth inversion for inland lakes. First, a water-depth inversion model is established by extracting the reflectance values of image bands using Rasterio, and the results are compared with a model built using reflectance values extracted with the commonly used GDAL method. Next, the water-depth inversion model is further optimized by combining the reflectance values of image bands extracted using Rasterio with remote sensing indices.

### 4.1. Model Performance Evaluation

**Figure 2:**
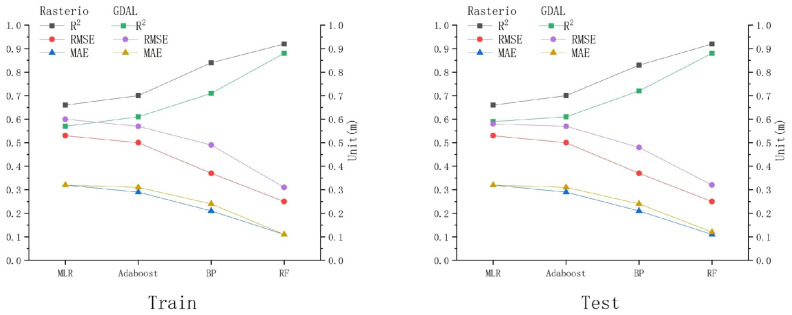
Performance evaluation of various models under GDAL and Rasterio.

Reflectance data were extracted using Python libraries such as GDAL and Rasterio, and the reflectance values obtained via both methods were used to calculate three accuracy metrics across various models for comparison and analysis. The goal was to identify the most suitable reflectance extraction method for remote sensing water-depth inversion in this study area. The accuracy performance of the models, as shown in Table 5 and Figure 2, was compared under GDAL and Rasterio.

Under Rasterio, the R^2^ values of the MLR model (R^2^> = 0.66, MAE = 0.32 m, and RMSE = 0.53 m), Adaboost (R^2^ = 0.7, MAE = 0.29 m, and RMSE = 0.50 m), BP model (R^2^ = 0.84, MAE = 0.21 m, and RMSE = 0.37 m), and RF model (R^2^ = 0.92, MAE = 0.11 m, and RMSE = 0.25 m) from the training set are always higher than or equal to those obtained from the testing set. Meanwhile, RMSE and MAE remain consistent, indicating that the overall correlation and accuracy of the fitted data under Rasterio are reasonable. In contrast, under GDAL, the R^2^ values of the MLR model (R^2^ = 0.57, MAE = 0.32 m, and RMSE = 0.58 m), Adaboost (R^2^ = 0.61, MAE = 0.31 m, and RMSE = 0.57 m), BP model (R^2^ = 0.71, MAE = 0.24 m, and RMSE = 0.49 m), and RF model (R^2^ = 0.88, MAE = 0.11 m, and RMSE = 0.31 m) from the training set are always lower than or equal to those from the testing set, while RMSE and MAE show the opposite trend. However, the maximum difference in R^2^, MAE, and RMSE between the training and testing sets for all models is 0.02, which also indicates that the overall correlation and accuracy of the fitted data are reasonable.

**Figure 3:**
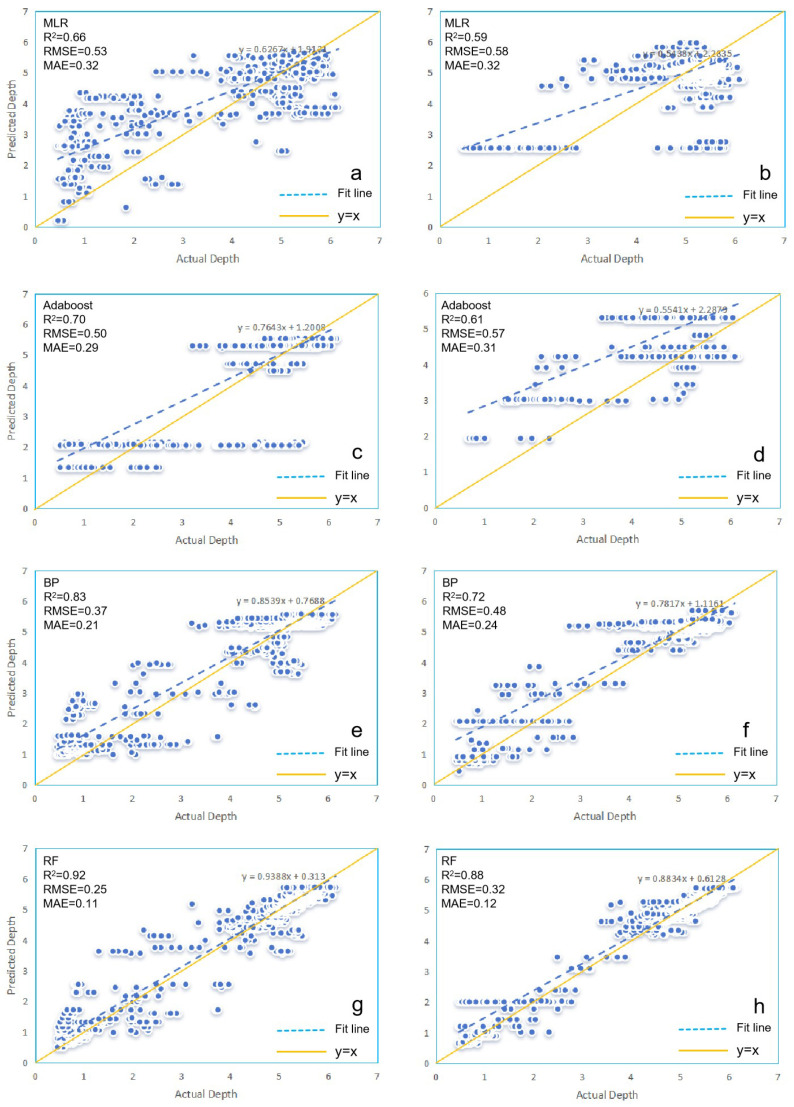
Model scatter plots: Rasterio (**a**,**c**,**e**,**g**) and GDAL (**b**,**d**,**f**,**h**).

Regardless of whether using Rasterio or GDAL, the three machine learning models consistently outperform the MLR model in terms of R^2^, RMSE, and MAE, demonstrating a stronger correlation between the simulated and measured water depths. Additionally, the RF model consistently has the highest R^2^ value, closest to 1, while RMSE and MAE are the smallest, both under Rasterio and GDAL. Moreover, as shown in Figure 3, there is a clear trend where the R^2^ values of the MLR model, Adaboost model, BP model, and RF model gradually increase, while RMSE and MAE decrease from Rasterio to GDAL. Models under Rasterio generally exhibit higher accuracies than those under GDAL.

The scatter plots in Figure 3 depict the predicted depths for the four models under Rasterio and GDAL. When compared with the measured water depths, the fitted lines generally exhibit an upward trend relative to the y = x line. However, differences among the models are evident in the degree of deviation from the y = x line, with increasing variability in the data points. This indicates variations in the predictive performance of the four models under Rasterio and GDAL. In the scatter plot for models under Rasterio, the RF model exhibits the smallest discrepancy between its fitted line and the y = x line, indicating superior prediction performance. For the MLR, Adaboost, and BP models, the fitted lines generally deviate from the y = x line. Specifically, within the 0–5 m depth range, the predicted water depths for these models (except Adaboost under GDAL) tend to be higher than the measured values, with notable errors in some instances. Conversely, for depths below 5 m, the trend reverses. Overall, the RF model under both Rasterio and GDAL has the fitted line closest to the y = x line, with similar scatter plots observed under both platforms. However, the deviation from the y = x line is greater in the GDAL scatter plot compared to Rasterio for all models, suggesting that the models under Rasterio are more suitable for water-depth inversion in this study.

As summarized above, the overall performance of the four models under Rasterio is superior to that under GDAL. Furthermore, the deviation between the fitted lines and the y = x line is smaller under Rasterio. Therefore, subsequent experiments will uniformly utilize the Rasterio tool for reflectance extraction in order to obtain more stable and reliable prediction results.

### 4.2. Model Optimization Analysis

**Figure 4:**
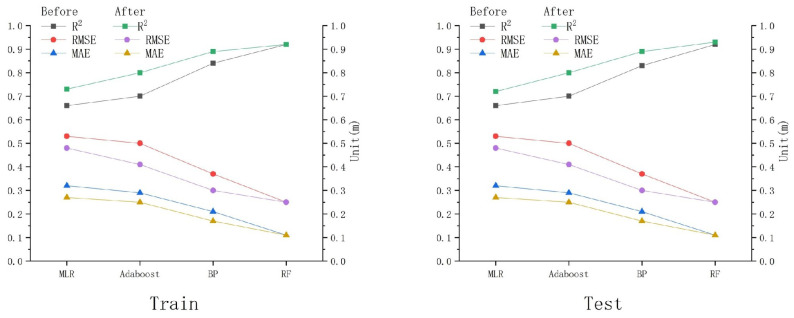
Accuracy before and after model optimization.

As shown in Table 6 and Figure 4, the optimized MLR (R^2^ = 0.73, MAE = 0.27 m, and RMSE = 0.48 m), Adaboost (R^2^ = 0.80, MAE = 0.25 m, and RMSE = 0.41 m), BP (R^2^ = 0.89, MAE = 0.17 m, and RMSE = 0.30 m), and RF models (R^2^ = 0.92, MAE = 0.11 m, and RMSE = 0.25 m) show R^2^ values from the training dataset that are typically higher than or equal to those obtained from the testing dataset, while RMSE and MAE remain consistent. This suggests that the overall predictive correlation and estimation accuracy of the fitted data for each optimized model are within acceptable ranges. Furthermore, the optimized MLR model (R^2^ = 0.72), Adaboost model (R^2^ = 0.80), and BP model (R^2^ = 0.89) exhibit higher R^2^ values compared to their pre-optimization counterparts, with corresponding decreases in RMSE and MAE. This suggests that the inclusion of remote sensing indices effectively improves the water-depth inversion accuracy of these three models. It is worth noting that although the pre- and post-optimization model accuracy of the RF model is higher than that of the MLR model, Adaboost model, and BP model, the R^2^, RMSE, and MAE values for the RF model remain highly consistent before and after optimization.

This consistency in performance may indicate that the RF model is inherently robust and less sensitive to the addition of new features, particularly in this context where remote sensing indices did not significantly alter its accuracy. In fact, while the addition of remote sensing indices could potentially enhance the performance of the RF model, the inherent robustness of the RF model may have mitigated the numerical significance of the observed improvement. For example, the R^2^ of the RF model increased by only 0.01 for the testing set after optimization.

**Figure 5:**
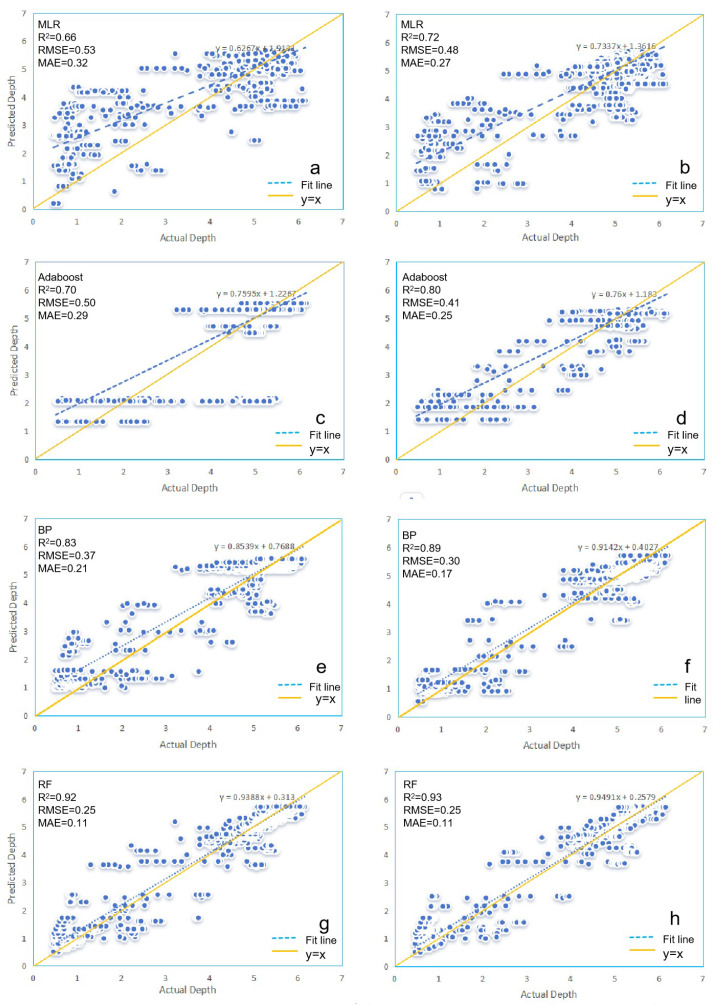
Scatterplot of the model: pre-optimization (**a**,**c**,**e**,**g**) and post-optimization (**b**,**d**,**f**,**h**).

To further compare and analyze the accuracy changes in various models, scatter plots are provided in Figure 5. These plots display the water-depth points from the testing set versus the measured water-depth points for each model in both their pre-optimization and post-optimization states.

After optimization, the MLR model showed an increase in R^2^ values from approximately 0.66 to 0.73 for both the training and testing sets, with RMSE and MAE values decreasing from 0.53 m to 0.48 m and from 0.32 m to 0.27 m, respectively. Prior to optimization, water-depth points within the 0–5 m range typically resided at the left of the y = x line, with the deviation of the fitted line increasing as the depth approached the surface (0 m). This phenomenon suggests that the water-depth inversion accuracy of the model before optimization was poor, partly due to significant terrain variations and the relatively low accuracy of the MLR model in shallow water areas. After optimization, the distribution of the measured and inverted water-depth points in the 0–5 m range became more uniform, with points evenly distributed on both sides of the y = x line. The water-depth points below 5 m also showed improved distribution. This indicates that the optimized MLR model has higher inversion accuracy than the unoptimized model, making it more suitable for water-depth inversion. In comparison to the MLR model, machine learning models offered superior accuracy in water-depth inversions across all depth ranges. In particular, the deviations between the fitted lines and the y = x line were smaller in the 0–5 m and >5 m depth ranges, and the predictions for the 0–5 m range were more precise. After optimization, the machine learning models showed more uniform distribution with respect to inverted and measured water depths on both sides of the y = x line, with fewer points deviating from the line. Additionally, the overall slope of the fitted line was lower than that of the y = x line.

Further analyses of Figure 5 reveal that for machine learning models, the inverted water depths for depths >5 m are greater than the measured depths, while for the 0–5 m range, the inverted water depths are smaller than the measured depths. In contrast, the MLR model only exhibits this pattern after optimization. This suggests that machine learning models are more suitable for water-depth inversion compared to traditional empirical models, and their application range is wider. Furthermore, after optimization, the RF model demonstrated the most closely aligned fitted line with the y = x line, indicating the best performance in water-depth inversion. The scatter plot before optimization was similar to that after optimization, but the deviation between the fitted line and the y = x line was larger before optimization, as was the case for the other three models. This indicates that the optimized models are more suitable for water-depth inversion in this study area compared to their pre-optimized counterparts.

In summary, generally, the accuracy of the four models after optimization is higher than before optimization. The deviation between the fitted lines and the y = x line is smaller for all four models after optimization. Consequently, the optimized models were used for water-depth inversion, and the RF model, which exhibited the highest accuracy after optimization, was selected as the final water-depth inversion model to obtain more stable and reliable prediction results.

### 4.3. Analysis of Bathymetric Estimation Results

**Figure 6:**
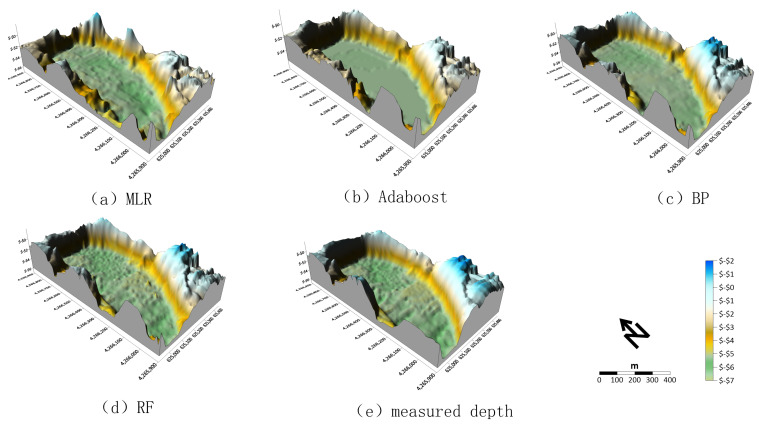
Three-dimensional topographic distribution map under Clearwater Lake (elevation in m and coordinates in m).

The geological structures of lakes, water quality assessment, and the prediction of future changes in lakes are closely linked to the lakebed’s lithology. Lakebed morphology is one of the key indicators used to characterize this type of lithology, as it not only reflects the sedimentary history and environmental evolution of the lake but also plays a crucial role in understanding lake dynamics and managing water resources. Three-dimensional water-depth data offer significant advantages in describing lakebed morphology. These data not only visualize the spatial distribution of water depths clearly but also capture subtle terrain variations with precision, providing a deeper and more comprehensive perspective for lake research. In stark contrast, two-dimensional water-depth maps, while providing basic depth information, lack the spatial dimensionality and intuitive terrain relief representation crucial for accurately depicting underwater topography, especially in revealing complex spatial water-depth distributions [34,35,36,37]. Therefore, to more precisely assess the water depth and terrain conditions of Qingshui Lake, this study employed the proposed approach to construct multiple models for water-depth inversion. The inverted water depths, along with the measured depths, were integrated as the third dimension to create a three-dimensional underwater topographic map, as shown in Figure 6.

Analyses of Figure 6 reveals significant differences between traditional water-depth inversion models (MLR model) and machine learning-based methods (Adaboost, BP, and RF). In the MLR model, the trends in the increasing water depths are relatively gradual from the edge to the center of the lake. In contrast, water depths inverted using machine learning models show a ’cliff-like’ increase, closely matching the measured water depths. This suggests that traditional methods are unsuitable for areas with sharp underwater topography changes, as they cannot capture the ’cliff-like’ depth increase. This limitation arises from the model’s insufficient linear fitting ability when dealing with complex terrain. Additionally, as Qingshui Lake is an artificial lake with a sharp depth increase from the shore towards the center, this results in a scarcity of sample data in the 0–5 m depth range, further compromising the model’s training performance. In contrast, the Adaboost, BP, and RF models exhibit superior performance in capturing detailed depth changes, reflecting underwater features with higher accuracy and aligning closely with measured water depths. These machine learning models are more adept at handling complex terrain variations and provide more detailed and precise water-depth change features. Notably, the RF model achieves high-precision inversion results across the lake, closely aligning with measured depths, indicating its superiority in simulating complex underwater environments.

Overall, there are significant differences between traditional water-depth inversion method and machine learning approaches. Traditional water-depth inversion methods do not perform well in complex underwater environments, such as lakes with dramatic changes in underwater topography. In contrast, machine learning models, particularly the BP and RF models, are capable of providing more detailed and accurate descriptions of the spatial distribution of underwater topography. The differences in model performance and the representation of underwater three-dimensional topography emphasize the necessity of selecting appropriate algorithms for water-depth mapping in specific regions. While traditional water-depth inversion methods may be sufficiently effective in areas with relatively stable underwater terrain, they struggle in regions with dramatic topographic changes. Machine learning methods, on the other hand, significantly outperform traditional water-depth inversion techniques in terms of accuracy and detail, and they are more capable of handling complex underwater terrain variations.

## 5. Discussion

### 5.1. Effect of Water Depth on Model Accuracy

**Figure 7:**
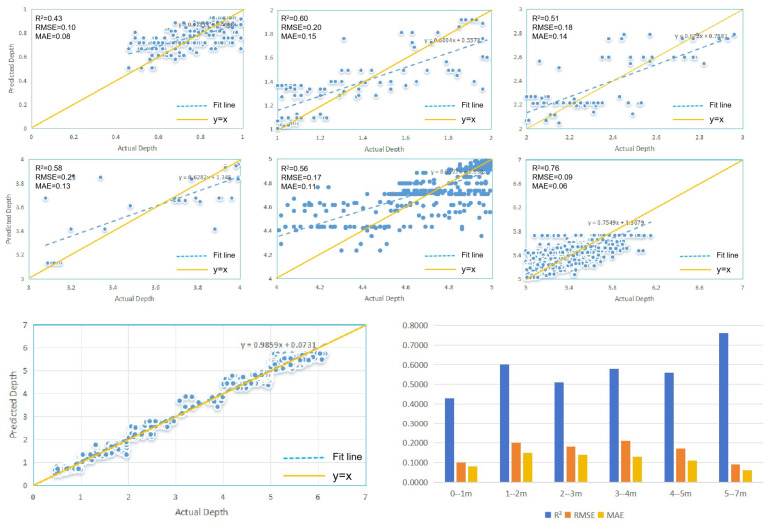
Scatterplot of predicted versus measured bathymetry for different profiles.

Through a comparative analysis of the predicted water depths from the optimized RF model and the measured water depths, it was found that the model overestimates water depths in the 0–5 m range and underestimates water depths in the 5–7 m range. To analyze this phenomenon in more detail, the 0–7 m water-depth range was divided into six different depth intervals, with the number of measurement points in each interval shown in Table 7. Since there are only 27 depth points in the 6–7 m range, the water depths greater than 5 m were treated as a separate depth interval for analyses. The R^2^, RMSE, and MAE for each depth segment were calculated, as shown in Figure 7.

Figure 7 clearly reveals a decline in the accuracy metrics of the optimized RF model across different depth intervals compared to the overall accuracy. Upon closer examination of the figure, particularly in the 0–1 m range, the R^2^ value drops to 0.43. Conversely, the depth interval with the highest water-depth inversion accuracy is the 5–7 m range, boasting an R^2^ value of 0.76. Further comparisons of the regression fitting line with the y = x line demonstrate a consistent pattern: Regardless of the depth interval, the model’s prediction accuracy exhibits a systematic trend where predicted values for larger depths are slightly lower than the measured values, while predicted values for shallower depths are slightly higher. This result aligns with the findings in Section 4.2, which highlighted similar discrepancies in model predictions based on sample distribution. The primary factor contributing to this phenomenon is the relatively low number of measured samples in the 0–5 m depth range, in contrast to the relatively higher number of samples in the 5–7 m depth range.

### 5.2. Effect of the Number of Model Factors on Model Accuracy

**Figure 8:**
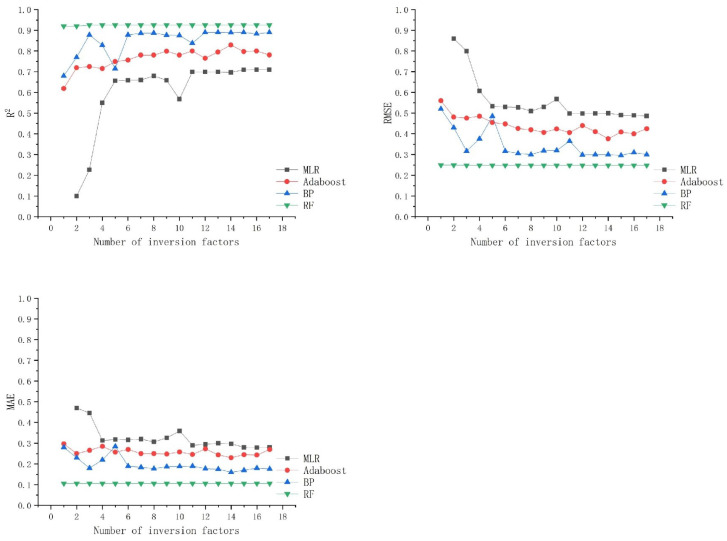
Effect of the number of estimation factors on modeling.

Whether using the MLR model or machine learning models, the number and quantity of inversion factors always limit the accuracy that the model can achieve and affect the inversion performance of the image’s model [16]. Therefore, using Python’s random sampling mechanism, an experimental scheme was designed: Starting with a single inversion factor, the number of factors was gradually increased to include all factors. These randomly selected factors were then introduced into the MLR model, Adaboost model, BP model, and RF model. To comprehensively evaluate the impact of the number of inversion factors on each model, three accuracy metrics were used as evaluation criteria. The results are shown in Figure 8.

Further analysis of the data presented in Figure 8, which illustrates the accuracy trends of various models with increasing inversion parameters, reveals that, even when using the same dataset, the accuracy metrics of different models vary significantly with changes in the number of inversion factors. However, as the number of inversion parameters gradually increases, the accuracy metrics of all models stabilize to some extent. Notably, the RF model demonstrates remarkable robustness, showing relatively little variation in performance in response to changes in the number of inversion parameters. In contrast, the MLR model, Adaboost model, and BP model are more significantly affected by changes in the number of inversion parameters until the number reaches seven. Beyond this point, the fluctuations in accuracy for these models diminish, and their performance tends to stabilize.

In practice, the selection and application of inversion factors are influenced by several factors. On one hand, they are constrained by the limitations of the spectral bands available in remote sensing imagery and the physical characteristics of light transmission through water. On the other hand, they are also affected by the diversity of methods for extracting reflectance from remote sensing imagery. As discussed, blindly increasing the number of inversion factors does not always lead to improved model inversion performance. Furthermore, the independence and non-collinearity of different inversion factors have a significant impact on the model’s accuracy [38]. Therefore, future research will focus on analyzing the effects of various inversion factors within different reflectance extraction methods on the inversion model. Specifically, we will consider the independence and collinearity of these factors in order to identify the optimal set of inversion factors for improving model performance.

## 6. Conclusions

This study innovatively incorporates remote sensing indices and Rasterio into a remote sensing water-depth framework, and performs a comparative analysis of the accuracy performance of various models using the GDAL reflectance extraction method. The main conclusions are as follows:

(1) Different remote sensing reflectance extraction methods can lead to a decrease in the accuracy of water-depth inversion. The machine learning models based on Rasterio, including RF, BP, and AdaBoost algorithms, and the MLR model, show better accuracy (R^2^ = 0.92, 0.83, 0.70, and 0.66; MAE = 0.11 m, 0.21 m, 0.29 m, and 0.32 m; RMSE = 0.25 m, 0.37 m, 0.50 m, and 0.53 m) compared to the same models based on the GDAL environment, which demonstrate lower accuracy (R^2^ = 0.88, 0.72, 0.61, and 0.59; MAE = 0.12 m, 0.24 m, 0.29 m, and 0.32 m; RMSE = 0.32 m, 0.48 m, 0.57 m, and 0.58 m).
(2) Compared to traditional experimental schemes, the water-depth inversion model based on Rasterio and integrated with remote sensing indices, constructed using this approach, shows varying degrees of improvement in accuracy (R^2^ = 0.93, 0.89, 0.8, and 0.72; MAE = 0.11 m, 0.17 m, 0.25 m, and 0.27 m; RMSE = 0.25 m, 0.30 m, 0.41 m, and 0.48 m). Notably, the RF model performs best in the 5–7 m water-depth range (RMSE = 0.09 m, MAE = 0.06 m), indicating that this model is more suitable for water-depth inversion in medium- to small-sized lakes.
(3) Future research should focus on optimizing reflectance extraction methods and assessing their impact on model performance. Enhancing model robustness through machine learning in diverse lake environments and integrating multi-source remote sensing data, such as high-resolution satellite and UAV imagery, will improve accuracy. Additionally, establishing a standardized water-depth inversion framework will facilitate the broader application of remote sensing in bathymetric studies.

## Figures and Tables

**Table 1:** Measuring instruments and accuracy.

Measuring Instruments	Instrument Model	Accuracy
GPS receiver	HUACE K50 (Shanghai Huace Navigation Technology Ltd., Shanghai, China)	Static plane accuracy: ±(3.0 mm + 0.5 ppm × D) Static elevation accuracy: ±(6.0 mm + 0.5 ppm × D)
Single beam	Nanjing Yuanhou FX160 (Nanjing Yuanhou Electronic Technology Co., Ltd., Nanjing, China)	1 cm ± 0.1% of depth (455 KHz: ±3 cm when depth < 0.7 m) (200 KHz: ±6 cm when depth < 1 m)
Unmanned boat	Type 130 single-hull unmanned boat (Wuhan Huawei Technology Co., Ltd., Wuhan, China)	

**Table 2:** Landsat 9 band reflectance information table.

Bands	Wavelength Range (μm)	Spatial Resolution (m)
Band 1 Coastal	0.433–0.453	30
Band 2 Blue	0.450–0.515	30
Band 3 Green	0.525–0.600	30
Band 4 Red	0.630–0.680	30
Band 5 NIR	0.845–0.885	30
Band 6 SWIR 1	1.560–1.660	30
Band 7 SWIR 2	2.100–2.300	30

**Table 3:** Correlation analysis between band reflectance and measured water depth under different reflectance methods.

Estimation Factors	Correlation	
	Rasterio	GDAL
B1	0.003	0.439
B2	0.105	0.533
B3	0.108	0.540
B4	−0.305	0.266
B5	−0.347	0.162
B6	−0.385	0.105
B7	−0.383	0.080

**Table 4:** Correlation analysis between estimation factors and measured bathymetry.

Estimation Factors	Correlation	Estimation Factors	Correlation
B1	−0.01	MNDWI1	0.68
B2	0.10	CDI	−0.25
B3	0.11	SDI	−0.26
B4	−0.30	NDMI	0.40
B5	−0.35	NDMI1	0.53
B6	−0.38	GI	0.25
B7	−0.38	GEMVI	−0.33
NDWI	0.68	VGCI	−0.52
MNDWI	0.69		

**Table 5:** Correlation analysis and error metrics for different models using Rasterio and GDAL.

	Models	Rasterio		GDAL	
		Train	Test	Train	Test
$R^{2}$	MLR	0.66	0.66	0.57	0.59
	RF	0.92	0.92	0.88	0.88
	Adaboost	0.70	0.70	0.61	0.61
	BP	0.84	0.83	0.71	0.72
RMSE (m)	MLR	0.53	0.53	0.60	0.58
	RF	0.25	0.25	0.31	0.32
	Adaboost	0.50	0.50	0.57	0.57
	BP	0.37	0.37	0.49	0.48
MAE (m)	MLR	0.32	0.32	0.32	0.32
	RF	0.11	0.11	0.11	0.12
	Adaboost	0.29	0.29	0.31	0.31
	BP	0.21	0.21	0.24	0.24

**Table 6:** Correlation analysis and error metrics for different models before and after some treatments.

Models		$R^{2}$		RMSE (m)		MAE (m)	
		Train	Test	Train	Test	Train	Test
MLR	Before	0.66	0.66	0.53	0.53	0.32	0.32
	After	0.73	0.72	0.48	0.48	0.27	0.27
RF	Before	0.92	0.92	0.25	0.25	0.11	0.11
	After	0.92	0.93	0.25	0.25	0.11	0.11
Adaboost	Before	0.70	0.70	0.50	0.50	0.29	0.29
	After	0.80	0.80	0.41	0.41	0.25	0.25
BP	Before	0.84	0.83	0.37	0.37	0.21	0.21
	After	0.89	0.89	0.30	0.30	0.17	0.17

**Table 7:** Number of points measured at different depths.

Bathymetric Interval	Average Water Depth (m)	Sample Size
0–1 m	0.77	706
1–2 m	1.34	361
2–3 m	2.33	248
3–4 m	3.66	124
4–5 m	4.73	1420
5–6 m	5.34	24,949
6–7 m	6.06	27

## Data Availability

Data are contained within the article.

## Abbreviations

The following abbreviations are used in this manuscript:

RF	Random forest;
BP	BP neural network;
MLR	Multi-band logarithmic ratio.
